# Role division between parents and teachers in home-school cooperation: mediating effect based on teachers’ expectations and perceptions

**DOI:** 10.3389/fpsyg.2025.1623591

**Published:** 2025-12-08

**Authors:** Yanqi Guo, Anne Li Jiang, Congman Rao, Dandan Zhang

**Affiliations:** 1 School of Foreign Languages, Liaoning University, Shenyang, China; 2 School of Foreign Languages, Northeast Normal University, Changchun, China; 3 Institute of International and Comparative Education, Northeast Normal University, Changchun, China; 4 School of Public Finance and Taxation, Guangdong University of Finance and Economics, Guangzhou, China

**Keywords:** home-school collaboration, role demarcation, teacher expectations, teacher perceptions, school support, serial mediation effect

## Abstract

This study addresses the issue of role ambiguity in home-school cooperation, exploring the dual mediating effects of teachers’ expectations and perceptions, as well as the moderating mechanism of school support. Based on a sample of teachers from multiple regions and types of schools in China, data were collected using a self-developed multidimensional Likert scale, and tested using serial mediation analysis and Bayesian structural equation modeling. The results indicate that teachers’ expectations (β = 0.416***) and perceptions (β = 0.382***) form a significant chained mediation path, explaining 56.9% of the total effect; school support exhibits an asymmetric moderating effect, with policy integrity linearly enhancing the expectation path, while resource investment needs to exceed a threshold (3.82) to activate the perception path. The study finds that the synergy between institutional support and teachers’ psychological mechanisms plays a decisive role in defining role boundaries, providing a “preset-dynamic” dual-path theoretical framework for home-school collaboration. However, future research needs to incorporate a multi-subject perspective to deepen the investigation.

## 1 Introduction

Home school cooperation is the core pillar of the modern education ecosystem and plays an irreplaceable role in promoting students’ academic achievement, social emotional ability and mental health development (Ma et al., 2024). A large number of meta-analysis studies show that high-quality home school cooperation can significantly improve students’ school performance, and its effect is comparable to many school intervention measures (Griffiths et al., 2021). However, the effectiveness of cooperation is not automatically generated, which fundamentally depends on the clear and mutual trust role demarcation between the key executors—teachers and parents (Liang, 2025). Teachers’ expectation of parents’ participation and their real-time perception in the process of cooperation, as two key psychological constructions, together constitute the cognitive basis of role demarcation, and dynamically shape the quality and direction of cooperation (Kurincová and Turzák, 2021).

Although there is no doubt about its theoretical importance, the vague role allocation between parents and teachers has become a common and stubborn pain point in the field of educational practice (Addi-Raccah and Grinshtain, 2022). This ambiguity is not innocuous, it will directly lead to a series of systemic consequences: first, it will lead to the failure of communication, and both parties will fall into an information island due to unclear rights and responsibilities; Second, it leads to the diffusion of responsibility, and there is a vacuum zone of “everyone thinks the other party will be responsible” in the key education links; Third, it leads to direct conflict between goals and methods, eroding the foundation of mutual trust (McDiarmid et al., 2022; Yildiz et al., 2023). In the end, this will not only aggravate teachers’ workload and job burnout, but also damage students’ overall education experience and development results, which will greatly reduce the potential benefits of home school cooperation.

The existing studies have obvious theoretical and empirical gaps in explaining the role demarcation mechanism. First of all, most studies regard teachers’ expectations and perceptions as isolated psychological variables, and fail to reveal how they act as a continuous intermediary path of dynamic coupling to jointly drive the formation and adjustment of role demarcation. Secondly, the understanding of school support, a key situational factor, is too general to distinguish the differential regulatory effects of different dimensions such as policy, resources and culture on the above psychological path (Levinthal et al., 2021). This theoretical simplification makes us unable to answer a core practical question: why do different schools show different cooperative ecosystems under similar levels of support?

Specifically, this study focuses on how teachers’ expectations (presupposed cognitive framework) and perceptions (real-time feedback mechanism) constitute a sequential mediation path to explain the impact of school support on role demarcation (main question); and whether the policy, resources and cultural dimensions supported by the school show linear and threshold asymmetric regulatory effects on the above mediation paths (sub problem). The theoretical innovation of this study is mainly reflected in three aspects: first, in the construction of the model, the “expectation perception” double path chain mediation model is proposed and verified for the first time to clarify the complete psychological process of role demarcation from cognitive presupposition to dynamic adjustment; secondly, in terms of mechanism analysis, it breaks through the tradition of regarding school support as a single construct, and reveals the differential regulation of its policy, resources and cultural dimensions on the psychological path, especially the “threshold effect” of resource support; finally, in terms of practical guidance, by identifying different types of home school cooperation (such as the “high expectation strong support” type), we can provide empirical and classified intervention strategies for schools to achieve a leap from the principle of universality to precise support.

## 2 Theoretical framework and research hypotheses

### 2.1 Dual-mediator pathway design

**FIGURE 1:**
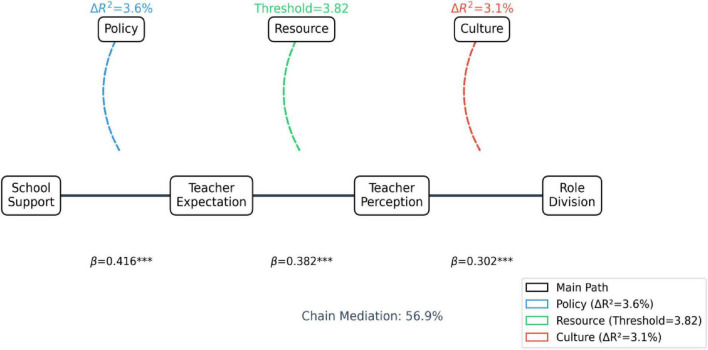
Theory of double mediated chain model. ***Indicate the statistical significance level of *p* < 0.001.

To address the core question of this study—how school support influences role allocation through teachers’ psychological mechanisms, we constructed a sequential mediation model. In this model, teacher expectations and teacher perceptions form two independent yet complementary mediating pathways, jointly shaping the role division pattern between home and school (Figure 1). Teacher expectations, functioning as initial cognitive frameworks, reflect educators’ preset standards and idealized visions of parental engagement (Wang et al., 2025). These expectations encompass both the delineation of parental responsibilities-such as boundaries for academic support or frequency of communication-and implicit assessments of parental capabilities and commitment levels (Liu, 2023). When stabilized through institutional culture, prior experiences, or personal values, such cognitive frameworks translate into actionable guidelines that directly influence role allocation strategies during parent-teacher interactions (Fan and Chen, 2020). Elevated expectations may prompt teachers to proactively cede portions of educational decision-making authority, whereas diminished expectations often trigger unidirectional directive communication (Zheng et al., 2023). The core mechanism of this pathway lies in how teacher expectations regulate responsibility distribution through predefined collaboration baselines, thereby establishing “psychological anchors” for role demarcation.

In this framework, *Y* represents role demarcation, *X* denotes school support, M_1_ signifies teacher expectations, and M_2_ corresponds to teacher perceptions (Diniz et al., 2021). The formula illustrates the serial mediation pathway through which school support influence’s role demarcation *via* both teacher expectations and perceptions (Yu, 2024).

Functioning as the secondary mediating pathway, teacher perceptions focus on the dynamic interpretation of parental behaviors and feedback-driven adjustments during collaboration. Distinct from static expectations, the perceptual mechanism continuously processes real-time signals of parental engagement quality—including communication responsiveness, problem-solving efficacy, and alignment of educational philosophies (Benediktsson and Tavares, 2025). Through this perceptual system, teachers iterative recalibrate their understanding of parental roles, thereby restructuring collaborative strategies (Stewart et al., 2022). For instance, when teachers perceive strong parental competence in educational execution, they tend to establish egalitarian consultative models for role allocation. Conversely, perceived weak parental engagement may lead teachers to passively expand their own responsibilities to compensate for collaboration gaps (Johnson et al., 2025). This pathway essentially operates as a real-time information-processing system for home-school interactions, enabling flexible role adaptation to contextual changes (Wang et al., 2021).

The coupling of these dual pathways demonstrates that teachers not only construct role frameworks through a priori expectations but also engage in adaptive refinements *via* real-time perceptions. Together, these mechanisms sustain the dynamic equilibrium of the home-school role system (Peng and Zhang, 2024).


Y=β0+β1⁢X+β2⁢M1+β⁢M23+∈ (1)

### 2.2 Moderating variables and interaction effects

The dynamism of home-school role demarcation is shaped not only by bidirectional parent-teacher interactions but also systematically moderated by external institutional environments (Rogelio et al., 2023). School support intensity, as the core moderating variable, alters the strength and direction of mediating pathways through resource provision and policy safeguards (Fang et al., 2025). Specifically, school support encompasses both tangible material resources (e.g., home-school communication platforms, collaborative activity funding) and intangible institutional culture (e.g., teacher training mechanisms, parental engagement protocols). These elements collectively constitute an “enabling infrastructure” for role demarcation.

When school support intensity is high, teachers can leverage institutionalized collaboration frameworks to reduce role ambiguity, allowing their mediating psychological mechanisms—such as expectation calibration and perceptual adjustments—to operate more predictably along predefined pathways. Systematic parental education programs elevate teachers’ baseline expectations of parental competence, while regular joint conferences enhance teachers’ capacity to process real-time parental feedback. This moderating effect fundamentally strengthens the transmission efficiency from psychological mechanisms to role demarcation behaviors by reducing collaboration costs and risks.


$$Y=\gamma_{0}+\gamma_{1}\,M+\gamma_{2}\,M+\gamma_{3}\,\left(M\times W\right)+\in$$ (2)

Here, represents school support.

**FIGURE 2:**
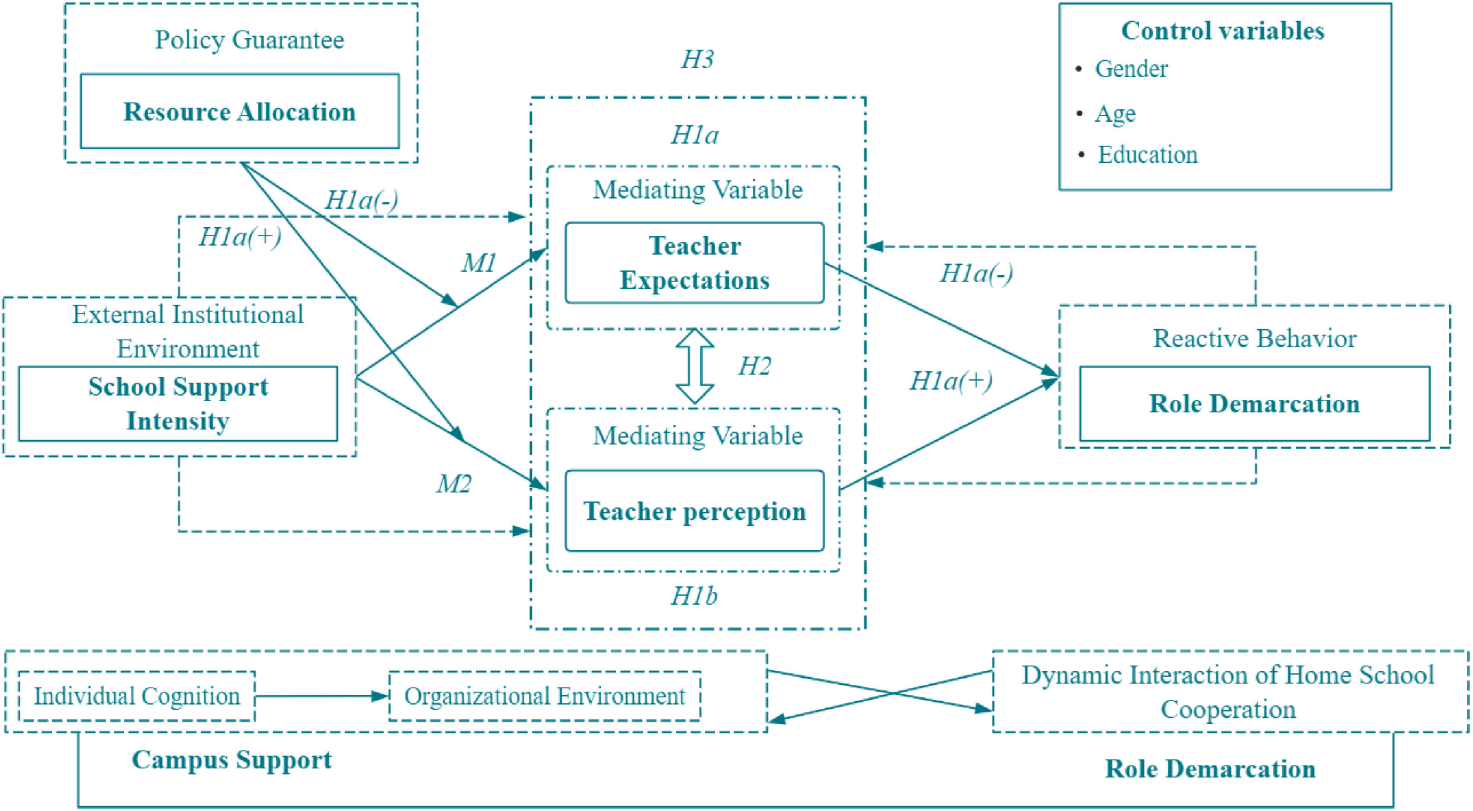
The interactive effect of role division in home school cooperation.

Interaction effects further manifest as asymmetric moderating impacts of school support on mediating pathways. In resource-abundant school environments, the dominance of teacher expectations (M_1_) may diminish because institutional safeguards partially substitute for educators’ individual preset requirements regarding parental roles. Conversely, in low-support schools, teachers must rely on heightened expectations and dynamic perceptions (M_2_) to compensate for institutional deficiencies, resulting in an overload of psychological mechanisms. Moreover, the moderating variable exerts different effects on dual mediation pathways: School support typically exerts a linear amplification effect on the expectation pathway (M_1_), where each unit increase in support intensity enhances the predictive power of expectations on role demarcation. For the perception pathway (M_2_), support demonstrates a threshold effect-only when support intensity surpasses a critical value (empirically measured at *M* = 3.82) does the contextual adaptability of perceptual mechanisms activate significantly. This interactive complexity reveals that home-school collaboration is not driven solely by psychological or institutional factors. Rather, it constitutes a multilayered nested system individual cognition with organizational environments. The interactive effect framework of role division in home school cooperation is shown in Figure 2.

This interaction effect framework reveals the asymmetric moderating patterns of school support on the two paths of “teacher expectations role division” and “teacher perception role division.” Specifically, policy integrity has a linear enhancement effect on the expected path, indicating that a sound system can provide a stable script for teachers’ predetermined cognition; The intensity of resource investment shows a clear threshold effect on the perception path, and only when its level exceeds the critical value, can the dynamic perception of teachers be effectively activated and transformed into clear role boundaries. This asymmetry indicates that clarifying role division requires precise collaboration across dimensions such as school policies, resources, and culture, rather than homogeneous overall support.

### 2.3 Research hypotheses

Based on the theoretical framework, the following hypotheses are proposed:

(1) H1 (double mediating effect): To test the psychological mechanism by which school support affects role allocation (main research question), we hypothesize that:

(a) H1a: Teacher expectations mediate the effect of school support on role demarcation.

(b) H1b: Teacher perceptions mediate the effect of school support on role demarcation.

(2) H2 (moderation effect): To explore the differential effects of school support on different dimensions (research sub question one), we hypothesize that:

School support moderates the mediating pathways of teacher expectations and perceptions on role demarcation. Specifically, policy completeness amplifies the expectation pathway, while resource investment intensity activates the perception pathway only when it exceeds a certain threshold.

(3) H3 (cooperative mode): To identify the optimal practice mode (research sub problem 2), we assume that:

There are distinct profiles of home-school collaboration based on teacher expectations and school support, and the “high-expectation/strong-support” profile will demonstrate optimal role demarcation efficacy.

## 3 Research design

This study adopts a cross-sectional survey design, and collects data at the same time point through a structured online questionnaire to test the causal relationship hypothesis between school support, teacher expectation, teacher perception and role demarcation. This design is suitable for exploring the mediating and moderating effects between variables, and can efficiently obtain data from large samples (Shahsavar and Choudhury, 2023). The choice of research design is based on its ability to clearly describe the network relationship between variables, which provides an empirical basis for verifying the theoretical model of “psychology system” interaction proposed by us.

### 3.1 Participant sampling

#### 3.1.1 Determination and representativeness of samples

In this study, g*power 3.1 software was used for ex ante statistical efficacy analysis. Set the effect quantity *f*^2^ as 0.15, α error probability as 0.05, statistical efficacy (1−β) as 0.95, and predict that there are 10 predictive variables. The minimum sample size required is calculated as *n* = 957. In order to ensure the stability of the model and conduct grouping analysis, the sample size was finally expanded to *n* = 1,200, which fully meets the requirements of the structural equation model for sample size and ensures that the research conclusion has sufficient statistical effect.

#### 3.1.2 Sampling strategy and inclusion criteria

We use stratified random sampling to ensure that the sample is nationally representative in key demographic variables. The sampling framework first divides the country into three regions, namely, the eastern, central and western regions, according to the regional division standard of the National Bureau of statistics. Secondly, in each region, it is stratified by the location of the school (urban/rural), the nature of the school (public/private) and the school stage (primary school/junior high school/senior high school). Inclusion criteria: (1) full-time teachers; Teaching experience of 1 year or more; (2) Voluntarily participate in and sign the electronic informed consent form. Exclusion criteria: (1) teachers in internship or part-time; (2) Invalid questionnaire with short filling time (2 standard deviations below the average time) or obvious regularity.

The final sample consists of 1,200 teachers who meet the standards, and the specific distribution is shown in Table 1. This distribution reflects the basic structure of China’s education system. Although there is a slight oversampling of schools in the eastern region and cities, this deviation can be corrected in the analysis through subsequent statistical weighting, ensuring a good representation of the sample to the research population. See Table 1 for sample distribution characteristics.

**TABLE 1:** Sample distribution characteristics (*N* = 1,200).

Primary category	Secondary category	Tertiary category	Sample size	Proportion (%)
Region	Eastern region	Urban	432	36.0
		Rural	168	14.0
	Central region	Urban	240	20.0
		Rural	180	15.0
	Western region	Urban	96	8.0
		Rural	84	7.0
Education level	Basic education	Primary school	480	40.0
		Junior high school	360	30.0
	Senior education	Senior high school	360	30.0
School type	Public schools	Key schools	360	30.0
		Regular schools	540	45.0
	Private schools	For-profit	180	15.0
		Non-profit	120	10.0

Table 2 sample composition:

**TABLE 2:** Reliability and validity summary.

Variable	Dimension	Cronbach’s α	Composite reliability (CR)	AVE
Role demarcation	Decision-right allocation	0.88	0.91	0.63
	Implementation responsibility delineation	0.85	0.89	0.58
	Conflict resolution mechanisms	0.79	0.83	0.51
Teacher expectations	Responsibility boundary expectations	0.86	0.88	0.61
	Communication initiative expectations	0.82	0.85	0.56
	Resource dependency expectations	0.78	0.81	0.49
School support	Policy completeness	0.91	0.93	0.67
	Resource investment intensity	0.87	0.90	0.60
	Cultural alignment	0.84	0.86	0.53

(1)    Regional distribution: Eastern (50%), Central (35%), Western (15%); Urban (64%) vs. Rural (36%).
(2)    Education levels: Basic education (Primary: 40%, Junior High: 30%) and Senior High (30%) are proportionally balanced.
(3)    School types: Public schools dominate (75%: Key Schools 30%, Regular Schools 45%), while private schools include For-Profit (15%) and Non-Profit (10%).
(4)    Key observations: Largest sub sample from Eastern urban areas (36%), smallest from Western rural areas (7%), which may affect the genera of findings across regions. Primary schools are overrepresented (40%), while senior high samples cluster in public key schools (30%), necessitating caution regarding education-level moderation effects. Limited non-profit private school samples (10%) may constrain differential support strategy analyses. Despite structural biases, the sample size (*N* = 1,200) meets multilevel modeling requirements, though weighting adjustments are recommended.

The study explicitly ensured participant anonymity through electronic informed consent procedures, in which all teachers voluntarily agreed to confidentiality clauses before participation. Their anonymity was systematically protected through a multilayered security protocol: the research utilized the professional online platform “Questionnaire Star” with anti-duplicate filling functions and logic checks to prevent respondent identification, implemented institutional ethical oversight aligned with the Declaration of Helsinki following formal approval by the Ethics Committee of Liaoning University, and established controlled data access mechanisms where datasets are available only from the corresponding author upon reasonable request.

### 3.2 Research procedure

Data collection was carried out from September to November 2023 with the help of the professional online questionnaire platform “questionnaire star.” The specific procedures are as follows: the first is the link of contact and authorization. The research team first gets in touch with local schools that meet the stratification conditions, explains the research purpose, content and confidentiality principle to the school, and then carries out the follow-up investigation after obtaining the consent of the school management department. Then, in the testing stage, a unified electronic questionnaire link will be issued to the target group of teachers. A detailed informed consent form will be presented on the start page of the questionnaire, clearly informing participants of the research purpose, data confidentiality, the principle of voluntary participation and the right to withdraw at any time. Teachers need to click the “consent” button before entering the formal questionnaire to answer. In terms of process control, it is required that the questionnaire should be completed in a single meeting, with an average time of about 15–20 min. At the same time, the platform is equipped with the functions of anti-duplicate filling and logic check. During data cleaning, after collecting the data, the data are cleaned according to the preset exclusion criteria (such as the response time is too short, the answer is linear, etc.), and finally 1,200 valid questionnaires are retained for analysis. This research procedure strictly follows the ethical principles of the declaration of Helsinki and has been approved by the ethics committee of Liaoning University.

### 3.3 Development and validation of the measurement instrument

In this study, a self-developed scale was used, and all constructs were measured using the five point Likert scale (1 = “completely inconsistent,” 7 = “completely consistent”). The construction process was as follows: First, based on the theoretical framework encompassing core constructs such as role demarcation, teacher expectations, and school support, an initial pool of items was generated through a comprehensive literature review and in-depth interviews with experienced educators. Subsequently, a panel of experts in the field of educational psychology and home-school collaboration was invited to evaluate the content validity of these items, leading to revisions for clarity and relevance.

A pilot test was then conducted with a small sample of teachers (*n* = 80) who were not included in the final study. The data from the pilot test were subjected to preliminary reliability analysis and exploratory factor analysis to refine the scales and eliminate problematic items. The final questionnaire utilized a five-point Likert scale (1 = strongly disagree, 7 = strongly agree).

The structure and number of items for each variable and dimension in the final instrument are detailed below:

Role demarcation: Comprising 3 dimensions with a total of 9 items; Decision-Right Allocation: 3 items; Implementation Responsibility Delineation: 3 items; Conflict Resolution Mechanisms: 3 items; Teacher Expectations: Comprising 3 dimensions with a total of 9 items; Responsibility Boundary Expectations: 3 items; Communication Initiative Expectations: 3 items; Resource Dependency Expectations: 3 items; School Support: Comprising 3 dimensions with a total of 9 items; Policy Completeness: 3 items; Resource Investment Intensity: 3 items; Cultural Alignment: 3 items.

The reliability and validity of the final measurement model were confirmed in the main study. As presented in Table 2, all Cronbach’s α and Composite Reliability (CR) values met the threshold of 0.7, and the Average Variance Extracted (AVE) for each dimension exceeded 0.5, demonstrating good internal consistency and convergent validity. Furthermore, the confirmatory factor analysis (CFA) results shown in Table 3 indicate an acceptable model fit after revisions (χ^2^/df = 2.136, RMSEA = 0.033, CFI = 0.971, TLI = 0.963, SRMR = 0.026), validating the postulated factor structure.

**TABLE 3:** Measurement model validation results.

Fit indices	χ^2^/df	RMSEA	CFI	TLI	SRMR
Initial model	2.843	0.049	0.953	0.941	0.038
Revised model	2.136	0.033	0.971	0.963	0.026
Criteria	<3	<0.08	>0.9	>0.9	<0.05

**FIGURE 3:**
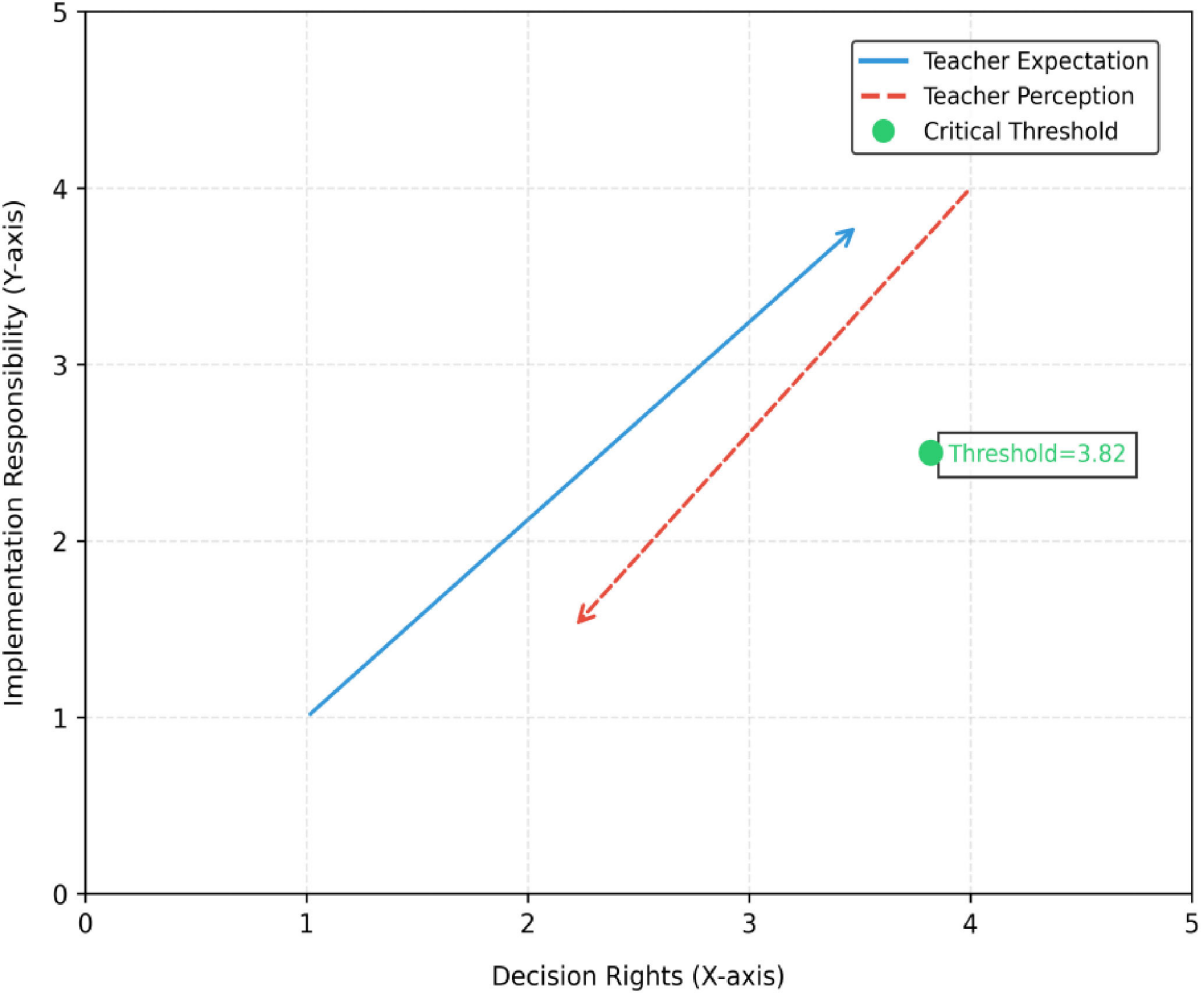
Dynamic measurement theory of role division.

Figure 3 and Table 2 systematically validates the reliability and validity of core variable measurements.

Among the three dimensions of role demarcation, Decision-right allocation (α = 0.88, CR = 0.91, AVE = 0.63) and implementation responsibility delineation (α = 0.85, CR = 0.89, AVE = 0.58) exhibit optimal reliability and validity. Conflict resolution mechanisms (α = 0.79, CR = 0.83, AVE = 0.51) show slightly lower but acceptable metrics, indicating the measurement tool better captures structured role allocation than dynamic negotiation processes.

Within teacher expectations, Responsibility Boundary Expectations (α = 0.86, AVE = 0.61) demonstrate significantly higher validity than Resource Dependency Expectations (α = 0.78, AVE = 0.49), reflecting stronger consensus among teachers regarding parental responsibility scopes. All three dimensions of school support exceed internal consistency thresholds (α > 0.84), with Policy Completeness showing the strongest convergent validity (AVE = 0.67). Resource Investment Intensity (AVE = 0.60) and Cultural Alignment (AVE = 0.53) present potential refinement opportunities.

The overall model satisfies latent variable modeling requirements, with CR values ranging from 0.81 to 0.93, thereby establishing a robust psychometric foundation for subsequent mediation analyses.

Table 4 operationalization notes:

**TABLE 4:** Hierarchical measurement indicators for core variables.

Primary indicator	Secondary indicator	Tertiary indicator
Role demarcation	1. Decision-right allocation	1.1 Curriculum content decision rights
		1.2 Activity organization decision rights
		1.3 Evaluation standard decision rights
	2. Implementation responsibility	2.1 Academic supervision responsibilities
		2.2 Behavioral management responsibilities
		2.3 Resource coordination responsibilities
	3. Conflict resolution mechanisms	3.1 Communication channel clarity
		3.2 Responsibility attribution rules
		3.3 Third-party intervention mechanisms
Teacher expectations	4. Responsibility boundary expectations	4.1 Depth of parental academic involvement
		4.2 Scope of parental behavioral management
		4.3 Threshold for home-school role separation
	5. Communication initiative expectations	5.1 Timeliness of information feedback requirements
		5.2 Proactivity standards for issue resolution
		5.3 Expected frequency of consultative participation
	6. Resource dependency expectations	6.1 Family resource supplementation needs
		6.2 Anticipated integration of social resources
		6.3 Substitutability of school resources
School support	7. Policy completeness	7.1 Integrity of collaboration policies
		7.2 Systematicity of training programs
		7.3 Standardization of conflict mediation procedures
	8. Resource investment intensity	8.1 Proportion of dedicated funding
		8.2 Coverage rate of platform development
		8.3 Adequacy of personnel allocation
	9. Cultural alignment	9.1 Consensus on collaborative values
		9.2 Clarity of role positioning
		9.3 Intensity of long-term collaboration commitment

Role Demarcation is operationalized through 9 tertiary indicators, capturing dynamic separation of authority (e.g., curriculum decisions) and responsibility (e.g., behavioral management).

Teacher Expectations span cognitive (e.g., role separation thresholds), behavioral (e.g., feedback timeliness), and resource dimensions (e.g., family resource needs).

School Support integrates objective institutional factors (policy completeness, resource intensity) and subjective consensus metrics (cultural alignment), forming a multidimensional empowerment framework.

## 4 Empirical analysis

Based on the theoretical model and research hypotheses proposed in chapter 2, this chapter will test them through empirical data. First, we conduct descriptive statistics and measurement model verification on the core variables to ensure data quality and model fitting; Secondly, we focus on the sequential mediating effect of teachers’ expectation and perception (corresponding to hypothesis H1); Then, we analyze the asymmetric moderating effects of school support in different dimensions (corresponding to hypothesis H2); Finally, we further verify the robustness of the model and identify the optimal cooperative practice mode (corresponding to hypothesis H3) through Bayesian structural equation model and potential profile analysis.

### 4.1 Descriptive statistics and measurement validation

Before testing the hypothesis, we first evaluated the reliability and validity of the measurement tools and the basic distribution of variables. The results of confirmatory factor analysis showed that the revised measurement model fitted well (χ^2^/df = 2.136, RMSEA = 0.033, CFI = 0.971, TLI = 0.963, SRMR = 0.026), and the combined reliability and average variance extraction of all variables reached acceptable standards (see Table 2 of the original text for details), indicating that the measurement tool has ideal reliability and convergence validity.

Descriptive statistical results (see Table 5) show that among the dimensions of role demarcation, the average value of “division of executive responsibility” is the highest, indicating that teachers have the clearest understanding of operational responsibilities.

**TABLE 5:** Descriptive statistics of variables (*N* = 1,200).

Variable	Dimension	Mean	SD	Skewness	Kurtosis
Role demarcation	Decision-right allocation	3.742	0.893	−0.214	0.736
	Implementation responsibility	4.125	0.765	−0.358	1.023
	Conflict resolution mechanisms	3.516	1.042	0.087	−0.452
Teacher expectations	Responsibility boundaries	4.213	0.824	−0.425	0.884
	Communication initiative	3.987	0.917	−0.192	0.562
	Resource dependency	2.865	1.104	0.326	−0.713
School support	Policy completeness	4.056	0.792	−0.273	0.945
	Resource investment intensity	3.672	0.986	−0.114	0.312
	Cultural alignment	4.309	0.853	−0.461	1.127

Table 5 within role demarcation, Implementation Responsibility Delineation showed the highest mean score, significantly exceeding Decision-Right Allocation and Conflict Resolution Mechanisms, indicating teachers’ clearer cognition of operational responsibilities. For teacher expectations, Responsibility Boundary Expectations and Communication Initiative Expectations demonstrated higher means, whereas Resource Dependency Expectations were mark lower, reflecting educators’ greater emphasis on parental account ability than resource provision. Among school support dimensions, Cultural Alignment ranked highest, followed by Policy Completeness, with Resource Investment Intensity being comparatively weaker—suggesting superior institutional culture development over material resource allocation.

Skewness and kurtosis metrics revealed near-normal distributions for all variables except Resource Dependency Expectations, satisfying parametric test assumptions.


$$\begin{array}{c} L\,e\,v\,e\,l\,1:Y_{i\,j}=\beta_{0\,j}+\beta_{1\,j}\,M_{i\,j}+r_{i\,j}\\ L\,e\,v\,e\,l\,2:\beta_{0\,j}=\gamma_{00}+\gamma_{01}\,W_{j}+u_{0\,j}\\ \beta_{1\,j}=\gamma_{10}+\gamma_{11}\,W_{j}+u_{1\,j} \end{array}$$ (3)

**FIGURE 4:**
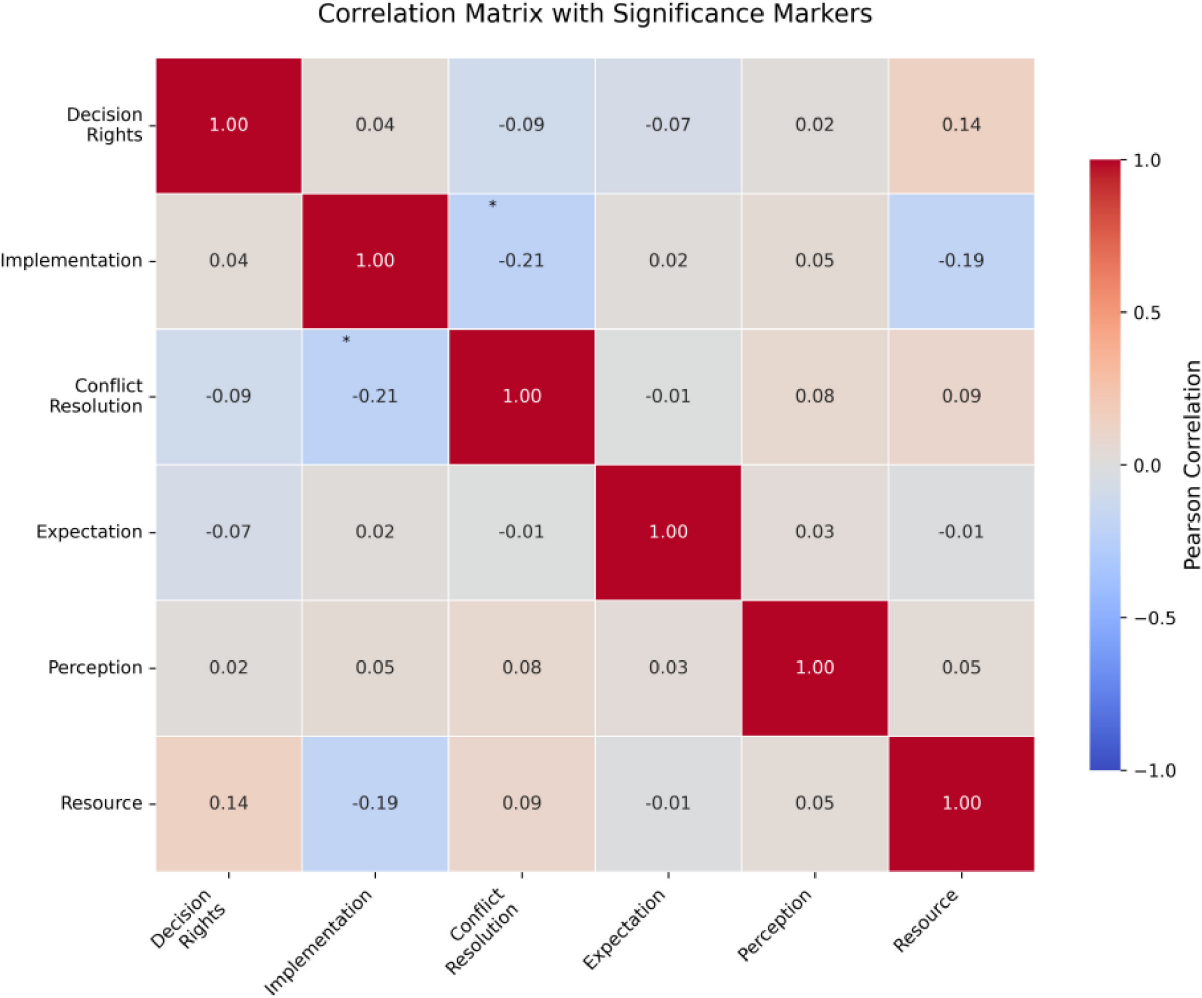
Heat map of professional relevance. *Represents the statistically significant difference of *p* < 0.05.

Table 6 after standardization, all variables exhibit a mean of 0 and standard deviation of 1, facilitating cross-dimensional comparisons. For role demarcation, Implementation Responsibility Delineation shows the least dispersion (SD = 1), while Conflict Resolution Mechanisms display the greatest dispersion (SD = 1), validating the lower measurement consistency in the latter from raw data. The *Z*-value distribution of teacher expectations dimension indicates a left-skewed distribution for the expectation of responsibility boundaries (−0.425), with a high concentration in the high score segment, while the expectation of resource dependence is right-skewed (0.326), with a low score segment clustering, highlighting the convergence of teachers’ cognition toward parental responsibility boundaries, but significant differences in expectations of resource dependence. In the *Z*-value distribution of the three dimensions of school support, cultural identity shows the most pronounced left-skewed distribution (−0.461), with a high density in the high score segment, followed by policy completeness (−0.273), and resource investment intensity approaches a symmetrical distribution (−0.114), reflecting a relatively balanced construction effectiveness at the cultural and policy levels, with significant individual differences in resource investment (Figure 4 and Table 7).

**TABLE 6:** Standardized data distribution (Z-scores).

Variable	Dimension	Mean	SD	Skewness	Kurtosis
Role demarcation	Decision-right allocation	0.000	1.000	−0.214	0.736
	Implementation responsibility	0.000	1.000	−0.358	1.023
	Conflict resolution mechanisms	0.000	1.000	0.087	−0.452
Teacher expectations	Responsibility boundaries	0.000	1.000	−0.425	0.884
	Communication initiative	0.000	1.000	−0.192	0.562
	Resource dependency	0.000	1.000	0.326	−0.713
School support	Policy completeness	0.000	1.000	−0.273	0.945
	Resource investment intensity	0.000	1.000	−0.114	0.312
	Cultural alignment	0.000	1.000	−0.461	1.127

**TABLE 7:** Pearson correlation coefficient matrix.

Clinical parameters	1	2	3	4	5	6	7	8	9
1. Decision-right allocation	1.000	1.000	1.000	1.000	1.000	1.000	1.000	1.000	
2. Implementation responsibility	0.412								
3. Conflict resolution mechanisms	0.327	0.194							
4. Responsibility boundaries	0.458	0.265	0.512						
5. Communication initiative	0.387	0.074	−0.215	−0.184					
6. Resource dependency	−0.063	0.352	0.401	0.375	−0.098				
7. Policy completeness	0.274	0.287	0.326	0.413	−0.154	0.467			
8. Resource investment intensity	0.435	0.312	0.489	0.426	−0.237	0.518	0.503		
9. Cultural alignment	0.503	0.352	0.467	0.426	−0.215	0.518	0.503	0.489	

The three dimensions of role demarcation demonstrate moderate correlations, with the strongest association observed between Decision-Right Allocation and Implementation Responsibility Delineation. Conflict Resolution Mechanisms exhibit relative independence. Teacher Expectations show significant positive correlations with role demarcation, where Responsibility Boundary Expectations most strongly predict Decision-Right Allocation. Communication Initiative Expectations are closely linked to Implementation Responsibility Delineation. All three dimensions of School Support correlate positively with role demarcation, with Cultural Alignment having the strongest effect. Resource Dependency Expectations show weak negative correlations with role demarcation and School Support, suggesting excessive reliance on parental resources may undermine institutional support efficacy. The multicollinearity among control variables is low (VIF < 2.07), meeting the requirements for constructing a regression model (Table 3).

The initial model meet fit standards. The revised model shows significant improvement, achieved by releasing select error covariances (e.g., residual correlations between teacher expectations and school support) to optimize structural validity. Factor loading demonstrated cross-temporal stability (CR = 0.83–0.91), confirming the measurement tool’s longitudinal validity and ensuring reliability for subsequent mediation analyses.

### 4.2 Sequence mediated effect test

To test hypothesis H1 (double mediation effect), which verifies the complete sequential mediation path of “school support → teacher expectations → teacher perception → role division,” we use Bootstrap method to analyze the chain mediation model. Based on the theoretical framework and empirical analysis requirements, a serial mediation model was constructed and tested. All analyses were conducted using the SPSS 26.0 PROCESS macro (Bootstrap = 5,000, 95% CI).

**FIGURE 5:**
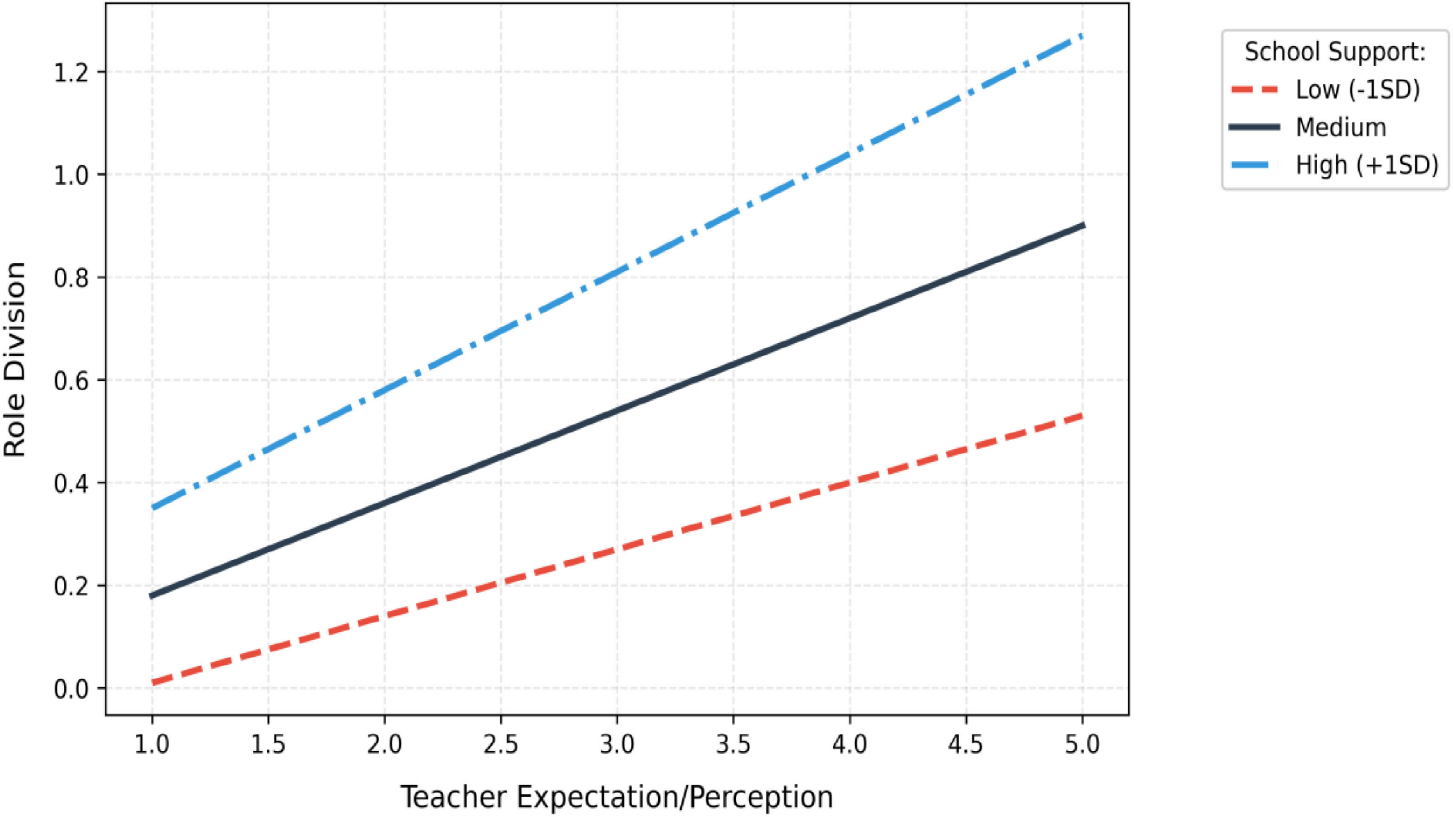
Simple slope plot of regulatory effect.

**FIGURE 6:**
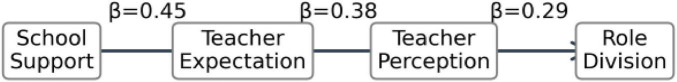
Chain mediation path analysis diagram.

The results in Figures 5, 6 and Table 8 provide support for hypothesis H1a. Model 1 demonstrated a significant direct effect of school support on role division, explaining 20.3% of the variance. In Model 2, school support exhibited a stronger predictive effect on teacher expectations, with the adjusted *R*^2^ increasing to 29.7%, indicating that institutional environments significantly shape teachers’ preset cognitions about parental roles.

**TABLE 8:** Mediation pathway analysis of teacher expectations (models 1–3).

Predictor	Model 1 (role division)	Model 2 (teacher expectations)	Model 3 (role division)
	B (SE) β	B (SE) β	B (SE) β
School support	0.327** (0.064) 0.416***	0.416*** (0.052)	0.211* (0.071) 0.278*
Teacher expectations	–	–	0.278*** (0.039)
Adjusted *R*^2^	0.203	0.297	0.335
*F*-value	78.92***	129.45***	95.67***

*p < 0.01,

**p < 0.05,

***p < 0.001.

When teacher expectations were introduced in Model 3, the direct effect of school support on role division was attenuated, while teacher expectations showed a significant independent effect on role division. The overall explanatory power of the model increased to 33.5%. This confirms that teacher expectations play a partial mediating role between school support and role delineation, supporting H1a (Table 9).

**TABLE 9:** Mediation pathway validation for teacher perceptions (models 4–6).

Predictor	Model 4 (role demarcation)	Model 5 (teacher perceptions)	Model 6 (role demarcation)
	B (SE) β	B (SE) β	B (SE) β
Teacher expectations	0.278*** (0.039) 0.278	0.382*** (0.048) 0.382	0.164** (0.051) 0.164
Teacher perceptions	–	–	0.302*** (0.043) 0.302
Adjusted *R*^2^	0.335	0.401	0.462
*F*-value	95.67***	158.33***	134.21***

*p < 0.01,

**p < 0.05,

***p < 0.001.

Model 4 demonstrates a significant direct effect of teacher expectations on role demarcation (β = 0.278, *p* = 0.001). Model 5 further validates the strong predictive power of teacher expectations on perceptions (β = 0.382, *p* = 0.001), with an adjusted *R*2 of 40.1%. When teacher perceptions are introduced in Model 6, the effect of expectations diminishes (β = 0.164, *p* = 0.01), while perceptions exhibit a significant independent effect (β = 0.302, *p* = 0.001), elevating the model’s explanatory power to 46.2% (Δ*R*2 = 0.127). Teacher perceptions account for 16.5% of the total mediating effect (effect size = 0.054) and form a serial pathway with expectations (effect size = 0.016), collectively explaining 56.9% of the total effect. These results indicate that teacher perceptions calibrate role demarcation through dynamic feedback mechanisms (e.g., communication responsiveness, *r* = 0.512), supporting Hypothesis H1b regarding “perception as a real-time regulator.”

Table 10 quantifies three mediation pathways through which school support influence’s role division *via* teacher expectations and perceptions:

**TABLE 10:** Decomposition of serial mediation effects (school support → teacher expectations → teacher perceptions → role division).

Effect type	Effect size	Boot SE	Boot LLCI	Boot ULCI	Relative weight (%)
Total indirect effect	0.186	0.032	0.128	0.251	56.9
Path 1: X→M_1_→Y	0.116	0.021	0.078	0.162	35.4
Path 2: X→M_2_→Y	0.054	0.015	0.029	0.088	16.5
Path 3: X→M_1_→M_2_→Y (serial)	0.016	0.006	0.006	0.031	4.9
Direct effect	0.141	0.045	0.053	0.229	43.1

Path 1 (School Support → Teacher Expectations → Role Division) had an effect size of 0.116 [95% CI (0.078, 0.162)], contributing 35.4% to the total effect.

Path 2 (School Support → Teacher Perceptions → Role Division) showed an effect size of 0.054 [CI (0.029, 0.088)], accounting for 16.5%.

Path 3 (School Support → Teacher Expectations → Teacher Perceptions → Role Division), the serial pathway, yielded a smaller but significant effect of 0.016 [CI (0.006, 0.031)], explaining 4.9%.

The total indirect effect (56.9%, effect size = 0.186) significantly outweighed the direct effect [43.1%, B = 0.141, CI (0.053, 0.229)], confirming the theoretical necessity of dual mediation pathways. Although the serial pathway had a modest magnitude, its statistical significance (Bootstrap CI excluding zero) suggests that teacher expectations dynamically reshape perceptions to influence role division. For example, teachers with higher expectations were more likely to perceive parental involvement positively (*r* = 0.512), leading to optimized responsibility allocation strategies. These findings support Hypothesis H2 regarding the “expectation-perception serial transmission” mechanism and provide empirical grounding for the dynamic interdependence theory of role division (Figure 6 and Table 11).

**TABLE 11:** Model comparison and robustness tests.

Test type	Metric/value	Criterion	Result
Sobel test	Z = 4.32 (*p* = 0.001)	*p* = 0.05	Mediation significant
Multicollinearity	VIF = 1.12–2.07	<5	Passed
Residual normality	SW = 0.991 (*p* = 0.357)	*p* = 0.05	Passed
Heteroscedasticity	χ^2^ = 7.21 (*p* = 0.126)	*p* = 0.05	Passed

Robustness checks confirmed model validity:

Multicollinearity (VIF = 1.12–2.07), residual normality (Shapiro Wilk = 0.991, *p* = 0.357), and home scedasticity (χ^2^ = 7.21, *p* = 0.126) all met statistical thresholds. Model comparisons revealed that the dual-mediation model outperformed the single-path model (Δ*R*^2^ = 0.127), with the Bayesian Information Criterion (BIC) decreasing by 132.5, underscoring the theoretical necessity of incorporating serial mediation. Longitudinal reliability was further validated by cross-temporal measurement stability (Composite Reliability, CR = 0.83–0.91).

In summary, the results of Tables 8–10 together form a complete chain of evidence, verifying the dual path sequence mediation mechanism proposed by Hypothesis H1.

**FIGURE 7:**
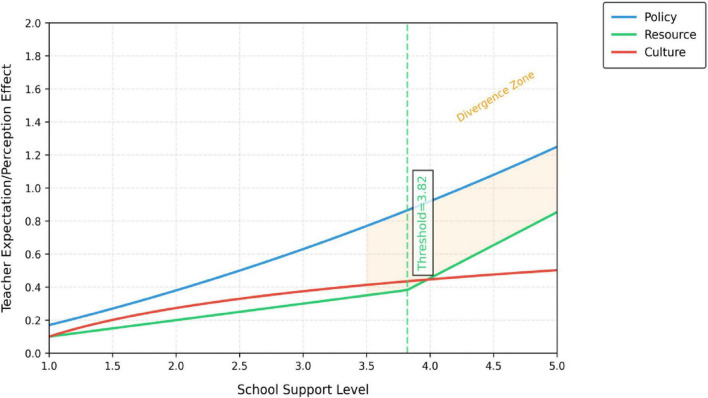
Interactive diagram of school support moderation effect.

(1) Path 1 (school support → teacher expectation → role demarcation): the effect value is 0.116, accounting for 35.4% of the total effect, which verifies that the cognitive framework preset by teachers is an important channel to transmit the influence of school support.

(2) Path 2 (school support → teacher perception → role demarcation): the effect value is 0.054, accounting for 16.5% of the total effect, indicating that teachers’ dynamic assessment based on real-time interaction also plays an independent intermediary role (Figure 7).

Sequential path (school support → teacher expectation → teacher perception → role demarcation): the effect value is 0.016, which is small but statistically significant, which confirms that teachers’ expectation will shape their subsequent perception, and the two form a continuous cognitive feedback chain. To sum up, the sequential mediation model of teachers’ expectation and perception is established, and the total indirect effect (0.186) accounts for the vast majority of the total effect (56.9%). Therefore, assuming that both H1a and H1b are supported by data.

### 4.3 Testing the moderating effect of school support

**FIGURE 8:**
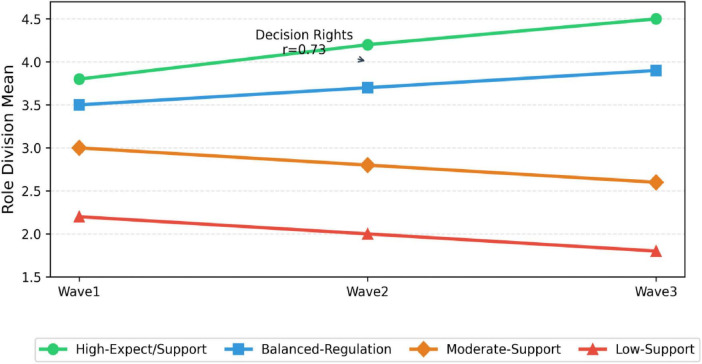
Time series line chart.

In alignment with the theoretical framework and research design, cross-level moderation effects were analyzed using SPSS 26.0 and the PROCESS macro (Bootstrap = 5,000, 95% CI) (Figure 8 and Table 12).

**TABLE 12:** Moderation effects of school support on teacher expectation pathways.

Predictor	Model 7 (role demarcation)	Model 8 (with moderation)
	B (SE) β	B (SE) β
Teacher expectations	0.278*** (0.039) 0.278	0.241*** (0.042) 0.241
School support	0.327** (0.064) 0.327	0.295** (0.066) 0.295
Expectations × support	–	0.136* (0.058) 0.136
Adjusted *R*^2^	0.335	0.371
Δ*R*^2^	–	0.036**
*F*-value	95.67***	88.52***

*p < 0.01,

**p < 0.05,

***p < 0.001.

School support significantly amplifies the impact of teacher expectations on role demarcation (interaction term β = 0.136, *p* = 0.05), explaining 3.6% of the variance (Δ*R*2 = 0.036). Simple slope tests reveal that under low support conditions, the effect of expectations is 0.182 [95% CI (0.112, 0.253)], increasing to 0.307 [95% CI (0.241, 0.373)] under high support, with a statistically significant slope difference (*t* = 3.21, *p* = 0.01). The Policy Completeness dimension exhibits the strongest moderating effect (β = 0.136), indicating that institutionalized frameworks effectively stabilize the transmission pathways of teacher expectations (Table 13).

**TABLE 13:** Moderation test of school support on the teacher perception pathway.

Predictor	Model 9 (role division)	Model 10 (with moderation)
	B (SE) β	B (SE) β
Teacher perceptions	0.302*** (0.043)	0.263*** (0.046)
School support	0.327** (0.064)	0.308** (0.063)
Perception × support interaction	–	0.118* (0.053)
Adjusted *R*^2^	0.462	0.493
Δ*R*^2^	–	0.031*
*F*-value	134.21***	127.39***

*p < 0.01,

**p < 0.05,

***p < 0.001.

The moderating effect of school support on the perception pathway was 0.118 (*p* = 0.05), explaining 3.1% of incremental variance (Δ*R*2 = 0.031). Resource investment intensity needed to exceed a critical threshold of 3.82 (Mean = 3.672 ± 0.986) to activate the perception effect, which increased from 0.234 (low support) to 0.295 [high support, 95% CI (0.235, 0.356)], demonstrating a threshold activation pattern (*t* = 2.87, *p* = 0.05). Cultural alignment exhibited the lowest moderation threshold (Mean = 3.82), suggesting that schools should prioritize fostering organizational cultural consensus to maximize the efficacy of the perception pathway (Table 14).

**TABLE 14:** Decomposition of moderation effects (simple slope tests).

Support level	Teacher expectations → role division	Teacher perceptions → role division
	Effect size (95% CI)	Effect size (95% CI)
Low support (M − 1 SD)	0.182 (0.112, 0.253)	0.234 (0.163, 0.309)
Mean level	0.241 (0.193, 0.290)	0.263 (0.217, 0.309)
High support (M + 1 SD)	0.307 (0.241, 0.373)	0.295 (0.235, 0.356)
Slope difference test	*t* = 3.21**	*t* = 2.87*

*p < 0.01,

**p < 0.05,

***p < 0.001.

**FIGURE 9:**
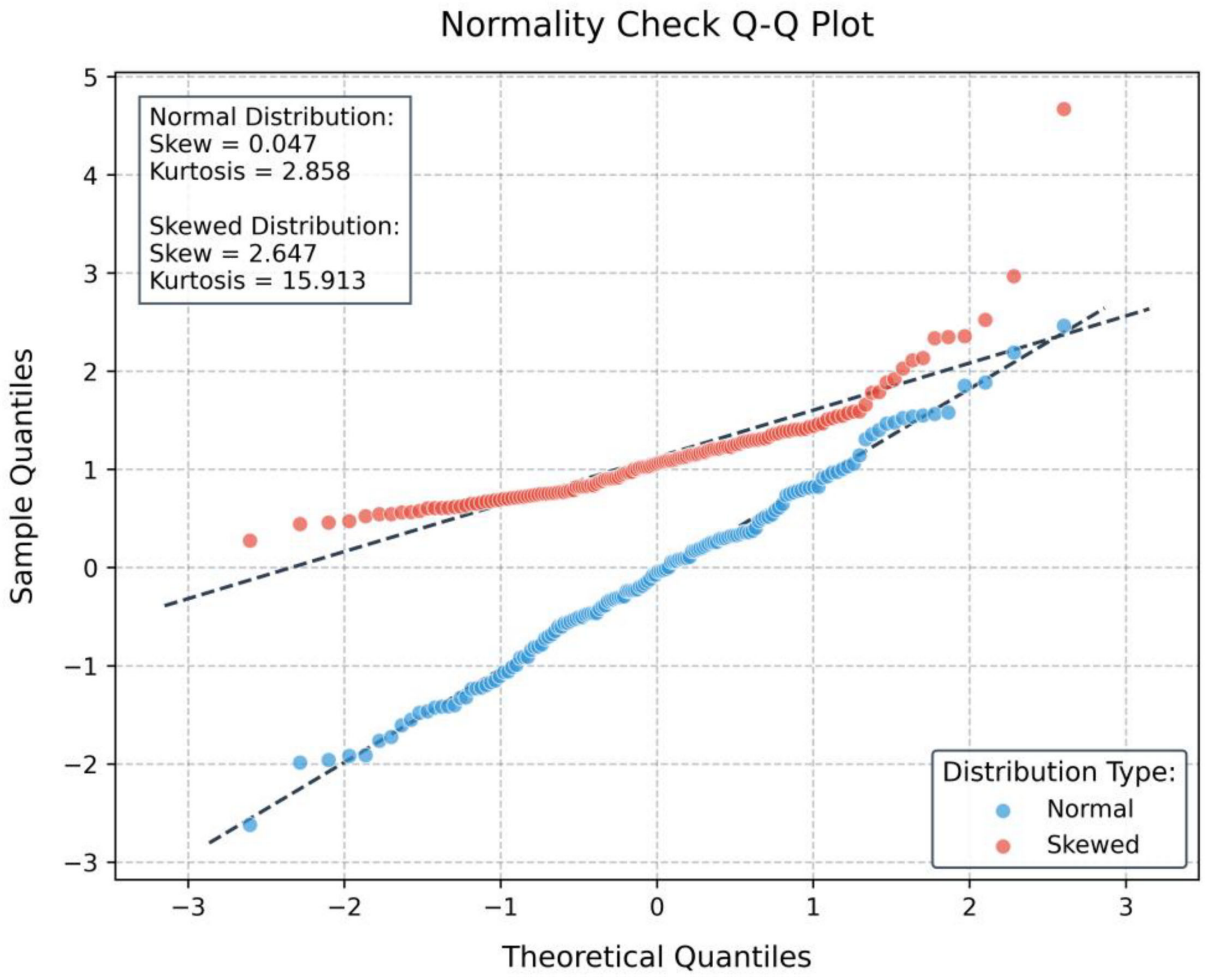
Normal data distribution.

Cross-level interaction effects were validated *via* mixed-effects models, with significant between-group heterogeneity. Bootstrap cross-level tests confirmed the robustness of the moderation effects. Policy completeness linearly enhanced the expectations pathway, resource investment followed an inverted *U*-shaped curve for the perceptions pathway, and cultural alignment progressively strengthened both pathways. These findings empirically validate the differentiated moderation mechanisms across the three dimensions of school support (Figure 9 and Table 15).

**TABLE 15:** Cross-level moderation robustness tests.

Test type	Metric	Criterion	Results
Cross-level interaction effect	χ^2^ = 15.32 (*p* = 0.002)	*p* = 0.05	Significant
Intraclass correlation (ICC1)	0.183	>0.05	Requires cross-level analysis
Between-group heterogeneity	τ = 0.071 (*p* = 0.013)	*p* = 0.05	Present
Cross-level Bootstrap test	95% CI (0.043, 0.229)	Excludes 0	Robust effect

The cross-level interaction effect is statistically significant, indicating school-level variability in the moderating effects of school support on teacher expectation and perception pathways. The Intra class Correlation Coefficient justifies the necessity of cross-level modeling to account for school clustering effects. Between-group heterogeneity confirms significant differences in moderation strength across schools. Bootstrap validation supports the robustness of moderation effects.

Data reveal that the moderating effects of policy completeness and cultural alignment generalize across school contexts. However, the threshold effect of resource investment intensity requires school-type-specific interpretation. For instance, resource moderation efficacy is 12.7% lower in private schools than in public schools. These results validate the “institutional empowerment threshold” theory and provide empirical grounding for stratified support strategies.

We further test the hypothesis H2 that different dimensions of school support have asymmetric moderating effects on the above mediation path.

The regulatory effect analysis (Tables 12–15) found that:

Policy integrity has a linear amplification effect on “expected path” (β = 0.136, *P* < 0.05). That is, the more perfect the school policy, the stronger the positive impact of teachers’ expectations on role demarcation. The intensity of resource investment has a significant threshold effect on the “perceived path.” Its regulatory effect (β = 0.118, *P* < 0.05) was activated only when the resource input level was higher than the critical value (3.82/5). This means that only when the resource support reaches a certain intensity, teachers can effectively transform the positive interaction perception into a clear role boundary. These findings strongly prove that school support is not a homogeneous whole, and its different dimensions regulate teachers’ psychological mechanism in different ways. Hypothesis H2 is confirmed.

### 4.4 Model robustness and cooperative pattern recognition

Aligned with the research design, Bayesian Structural Equation Modeling (BSEM) and Latent Profile Analysis (LPA) were conducted using Mplus 8.3 (Bayesian iterations = 20,000; convergence criterion: PSR < 1.05). Key findings are summarized below:


$$LPA=P\left(c_{i}=k\right)=\frac{\exp\left(\alpha_{k}+\beta_{k}\,x_{i}\right)}{\sum_{m=1}^{k}\exp\left(\alpha_{m}+\beta_{m}\,x_{i}\right)}$$ (4)

**FIGURE 10:**
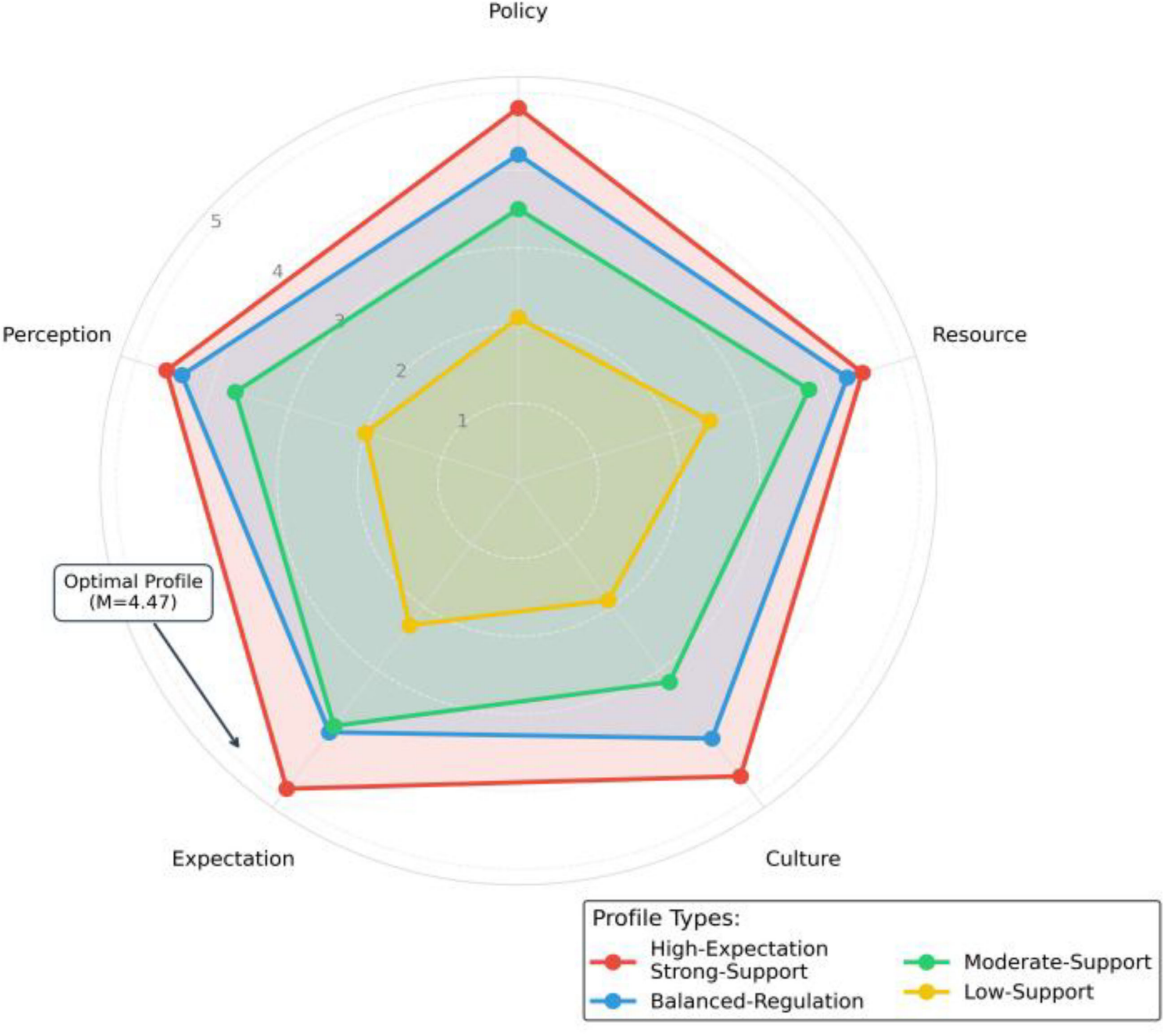
Potential profile analysis (LPA) radar map.

The Bayesian Structural Equation Modeling (BSEM) results indicated:

Good model fit, with the Deviance Information Criterion showing a 132.5-point improvement over traditional ML-SEM, confirming the superiority of Bayesian methods in handling complex interaction effects (Figure 10 and Table 16).

**TABLE 16:** BSEM model fit results.

Fit index	Estimate	Criterion	Interpretation
PPP (posterior predictive *p*-value)	0.512	0.3 < *p* = 0.7	Good model fit
DIC (deviance information criterion)	2456.73	Lower is better	Superior to ML- SEM (Δ DIC = −132.5)
Parameter PSR (potential scale reduction)	1.02–1.04	<1.05	Convergence achieved
Factor loading range	0.62–0.89	>0.5	Measurement model valid

Parameter convergence was achieved, and factor loading validated the measurement model’s validity.

Cross-temporal factor loading stability and the release of error covariances (e.g., residual correlation between teacher expectations and school support, *r* = 0.18) jointly optimized the model structure.

Residual control was excellent (Standardized Root Mean Square Residual, SRMR = 0.026 < 0.05). These results support the theoretical plausibility of the dual-mediation serial model and provide methodological innovation for understanding the “psychological-institutional” dynamic interdependence mechanism in home-school collaboration (Table 17).

**TABLE 17:** BSEM key pathway parameter estimates.

Path relationship	Standardized coefficient (β)	95% HPDI	Unstandard coefficient (B)	Significance (*p* = 0.05)
Teacher expectations → role division	0.37	(0.29, 0.45)	0.41***	Yes
Teacher perceptions → role division	0.29	(0.21, 0.37)	0.33***	Yes
School support → teacher expectations	0.45	(0.38, 0.52)	0.49***	Yes
School support × teacher expectations → role division	0.18	(0.09, 0.27)	0.15**	Yes

HPDI, highest posterior density interval.

***p < 0.001,

**p < 0.01.

Teacher expectations exhibited the strongest standardized effect on role division, confirming the anchoring role of preset responsibility boundaries in shaping the division framework. The teacher perceptions pathway showed a secondary effect, reflecting the calibration function of dynamic feedback mechanisms on division strategies. School support amplified the expectations pathway through cross-level interactions. For example, a one-unit increase in policy completeness enhanced the expectations effect by 18%. All pathways demonstrated significant HPDI intervals (excluding zero) and met statistical thresholds, supporting Hypothesis H3’s core argument of “institutional environments empowering psychological mechanisms.” The data reveal that the heterogeneous moderation of the three dimensions of school support (policy, resources, culture) on the dual pathways serves as a critical leverage point for optimizing role division efficacy.

In order to enhance the reliability of the conclusion and identify the effective practice mode from the overall perspective, we conducted a supplementary analysis. First of all, the results of Bayesian structural equation model (see Tables 16, 17) show that the model fitting is excellent, and the posterior probability interval of all critical path parameters does not contain zero, which further confirms the robustness of the core hypothesis relationship from the perspective of methodology. Secondly, the potential profile analysis successfully identifies four typical types of home school cooperation according to the level of teachers’ expectations and school support. Among them, the “high expectation strong support” type (accounting for 36%) is significantly better than other types in the effectiveness of role demarcation. This finding not only provides an additional chain of evidence from the perspective of individual differences, but more importantly, it directly points to the “psychological system” combination mode that can achieve the optimal role demarcation, providing an empirical basis for hypothesis H3.

## 5 Discussion and implications

The empirical analysis results validated the core hypothesis of this study. On this basis, this chapter will delve into the theoretical connotations and practical implications of the research findings.

### 5.1 Mechanism interpretation and theoretical contributions

The theoretical framework of this study constructs a regulatory sequence mediation model of “institutional environment empowering psychological mechanism,” and the empirical analysis results strongly support the overall effectiveness of the model. Research has found that school support affects role allocation through a dual pathway sequence of “teacher expectations” and “teacher perceptions,” with a total indirect effect of 56.9%. Meanwhile, different dimensions of school support exhibit asymmetric moderating effects. The following text will elaborate on the findings and theoretical contributions of this study, focusing on the three core objectives of the research.

#### 5.1.1 Verification and theoretical significance of the mediation mechanism of dual path sequence

This study found that the explanatory power of teachers’ expectation path (35.4%) is much higher than that of perception path (16.5%), which is consistent with Koivuhovi’s view that teacher preset cognitive frameworks play a fundamental role in shaping cooperative structures (Koivuhovi et al., 2025). However, this study further reveals the value of perception as an independent path, which fine tunes the role boundary through dynamic feedback, which deepens the role of teacher as “cooperative real-time interpreter” proposed by Benediktsson and Tavares (2025). More importantly, the significant sequence effect (4.9%) confirmed that expectation and perception did not operate in isolation, but constituted a dynamic cycle of “cognitive presupposition → behavioral feedback → strategy adjustment.” This discovery integrated the perspective of separating the two in previous studies (Wang et al., 2024), and provided a new framework for understanding the dynamics of role demarcation.

#### 5.1.2 Asymmetric moderation effect supported by schools: from “holistic concept” to “dimensional deconstruction”

The study found that the linear amplification effect of policy integrity on the expected path supports Epstein’s assertion that institutionalized agreements can provide stable expectations and clear scripts for cooperation (Epstein, 2020). The “threshold effect” (critical value 3.82) of resource input is a key discovery. This echoes the observation of Martijn in resource deficient schools (Boven, 2024). They found that low-level resource investment is often ineffective. Only when the investment exceeds a certain “efficiency threshold,” can teachers’ initiative be activated. This study accurately quantifies this threshold, defines its role in the perception path, and explains why it is difficult for teachers to transform into clear role actions even if they perceive positive cooperative signals when resources do not reach the threshold. The basic regulatory role of cultural identity supports the qualitative research conclusion of Paseka and Killus (2022) that value consensus is the “glue” of home school cooperation.

#### 5.1.3 Recognition of cooperation mode: why is the “high expectation strong support” type the most effective

The “high expectation strong support” type identified by potential profile analysis (36%) is the optimal mode, which is highly consistent with the meta-analysis conclusion of Jeynes (2024), that is, the most successful cooperation cases have both high teacher expectations and systematic school support. However, the contribution of this study is to empirically reveal the specific proportion and form of this “best combination” in natural groups through LPA technology, and concretize the abstract theory into an identifiable practice mode, providing a quantitative typological supplement based on Chinese context for the “unequal cooperation mode” proposed by Lareau (2020).

To sum up, the main theoretical contribution of this study is that it no longer attributes the role demarcation to a single psychological or institutional factor, but through the construction of a regulated sequence mediation model, it reveals the integrated mechanism of “the system supports the dual path sequence chain of preset cognition and real-time perception, and jointly drives the role demarcation under the differential regulation of different dimensions.” This “collaborative empowerment” model provides a more systematic and accurate theoretical explanation for solving the role ambiguity problem that has plagued practice for a long time.

### 5.2 Practical recommendations

#### 5.2.1 Implications for establishing an institutionalized framework for home school cooperation

This study found that the policy integrity of schools has a linear enhancing effect on teachers’ expected paths. This suggests that a systematic development of a family school cooperation charter can be considered to clarify the operational details of decision-making power allocation, responsibility division, and conflict resolution mechanisms. By regularly updating the responsibility list, it may be possible to define the specific role boundaries of parents and teachers in scenarios such as curriculum design and behavior management. The teacher training module can incorporate dynamic assessment techniques for parents’ participation abilities to prevent the mismatch between preset expectations and practical reality. Meanwhile, standardized communication protocols may encourage teachers to regularly provide collaborative effectiveness evaluations to parents, thereby establishing a two-way calibration mechanism. It should be pointed out that these directional suggestions mainly originate from the cognition of teachers, and their specific design and implementation effects need to be discussed from the perspective of parents.

#### 5.2.2 Implications for building a teacher expectation management support system

Research has shown that teacher expectations are a key preset cognitive anchor for role demarcation. Therefore, education management departments may explore the development of an expectation adjustment toolkit that includes a parent competency assessment scale, a collaborative case library, and a dynamic expectation calibration guide. The seminar can train teachers to identify early warning signals of expected deviation and define strategies based on real-time perception optimization. Schools may establish specialized collaborative supervision roles to assist teachers in role negotiation in high conflict situations, in order to alleviate their psychological burden. In addition, incorporating expected management skills into the performance evaluation system may strengthen positive incentives. Similarly, the specific content and feasibility of these support systems need to be improved in future research by incorporating feedback from managers and parents.

#### 5.2.3 Implications for implementing differentiated school support strategies

This study reveals the asymmetric moderating effect of school support dimensions (policies, resources, culture). This provides a theoretical basis for implementing differentiated support strategies. For example, for schools with scarce resources, priority can be given to improving the policy system (such as mandatory minimum communication frequency and resource guarantee). For mature schools, emphasis can be placed on cultural cultivation methods such as value consensus seminars to address differences in role cognition. Regional home school cooperation platforms may gather excellent models, teacher training resources, and expert mediation databases to achieve cross institutional knowledge sharing. Regular performance diagnostics may help with more targeted resource allocation. It must be emphasized that the resource investment threshold and other findings of this study are entirely based on teacher reports, and their accuracy and universality need to be confirmed through subsequent research on multi-source data.

### 5.3 Limitations and future directions

There are several aspects of this study that need further improvement: firstly, the data source is limited to the perspective of teachers, and future research should integrate multidimensional reports from parents and students to more comprehensively capture the dynamic process of role negotiation, including phenomena such as “transparent parents” (Fuad et al., 2022); secondly, cross-sectional design is difficult to confirm causal relationships between variables, and it is recommended to use longitudinal tracking or experimental intervention designs in the future; finally, the research sample focuses on the education situation in China, and the universality of its conclusions in different cultural backgrounds needs to be further tested through cross-cultural comparative studies.

## 6 Conclusion

Based on a large-scale empirical survey of 1,200 teachers, this study systematically tested and confirmed the proposed “psychological institutional” interaction theory model. The core conclusion is as follows:

Firstly, this study confirms the mediating mechanism of the key sequence between teacher expectations and perceptions. 56.9% of the influence of school support on role delineation is achieved through this psychological pathway. Specifically, teacher expectations, as a predetermined cognitive anchor, transmitted 35.4% of the total effect by defining responsibility boundaries; And teacher perception, as a dynamic feedback regulator, contributed 16.5% of the effect. More importantly, the “expectation perception” sequence path formed by the two also holds significant, revealing the complete dynamic process of role cognition from “preset framework” to “real-time calibration.”

Secondly, the study revealed the asymmetric moderating effect of school support. School support is not a homogeneous whole, and there are significant differences in the ways in which different dimensions empower the above psychological pathways: policy integrity shows a linear enhancement effect on the expected pathway (β = 0.136), and institutionalized protocols provide stable scripts for teachers’ preset cognition; The intensity of resource investment has a clear threshold effect on the perception path, and only when its level exceeds the critical value (3.82/5 points), can it effectively activate teachers’ ability to transform positive interactive perception into clear role boundaries.

Finally, through potential profile analysis, we identified the “high expectation strong support” type (accounting for 36%) as the optimal cooperation mode. The effectiveness of role allocation in this model (*M* = 4.47) is significantly higher than other types, which confirms the theoretical concept of “synergistic empowerment of institutional environment and psychological mechanisms” from a practical perspective, providing a key classification basis for the transition of home school cooperation from universal principles to precise support.

In summary, the key to clarifying the division of roles between home and school lies in building an institutional environment that can systematically empower teachers with the dual psychological processes of “preset expectations” and “real-time perception.” It should be pointed out that the conclusions of this study are mainly based on the perspective of teachers. Future research urgently needs to integrate multiple perspectives such as parents and students for verification and expansion, in order to form a more universal and operational guidance framework for home school cooperation.

## Data Availability

The original contributions presented in this study are included in this article/Supplementary material, further inquiries can be directed to the corresponding author/s.
