# Supplementary material for: Role division between parents and teachers in home-school cooperation: mediating effect based on teachers’ expectations and perceptions
Source: Front Psychol. 2025 Dec 8;16:1623591. doi: 10.3389/fpsyg.2025.1623591 (PMC12723000; doi:10.3389/fpsyg.2025.1623591)
Supplement: Supplementary file 3 [file Supplementary_file_1.docx]

1. Scale Validation Rigor Beyond Core Psychometrics

While the main paper reports Cronbach’s alpha and confirmatory factor analysis (CFA) for scale validity, supplementary analyses included additional steps to ensure the measures’ robustness. First, we conducted split-sample cross-validation: 600 teachers (50% of the sample) were randomly assigned to an exploratory factor analysis (EFA) group, and the remaining 600 to a CFA group. The EFA (principal axis factoring with varimax rotation) extracted 3 factors for each construct (Role Demarcation [RD], Teacher Expectations [TE], School Support [SS])—explaining 68% of variance for RD, 72% for TE, and 70% for SS—with all items loading >0.5 on their intended factors. The CFA on the holdout sample confirmed the 3-factor structure for each construct (CFI>0.95, RMSEA<0.06, SRMR<0.05), consistent with the main paper’s results. Second, we used item response theory (IRT) to evaluate the Likert scale items: the Rasch model revealed that items for each subdimension had appropriate difficulty (range: -1.2 to 1.1) and discrimination (range: 0.8 to1.5) parameters, indicating they effectively differentiated between teachers with varying levels of the construct. Finally, test-retest reliability was assessed with 50 teachers who completed the questionnaire twice (2-week interval): intraclass correlation coefficients (ICC) for composite scores were 0.87 for RD,0.89 for TE, and0.91 for SS—confirming the scales’ temporal stability. These steps ensure the measures are valid, reliable, and suitable for cross-group comparisons.

2. Stratified Sampling: Detailed Stratum Design & Representativeness

The main paper mentions stratified random sampling but does not elaborate on the stratum-specific design. To ensure representativeness, we stratified the sample by three nested criteria: region (Eastern/Central/Western), school type (public/private), and urban/rural location. For each region: we selected 10 schools (5 public,5 private) from urban and rural areas (5 each), resulting in 30 schools total. Within each school, we randomly selected teachers using official rosters—ensuring no self-selection bias. Power analysis for each stratum confirmed that we had sufficient sample size: for the Eastern region (50% of the sample,600 teachers), we could detect small effect sizes (d=0.2) with 95% power; for the Western region (15%,180 teachers), we could detect medium effect sizes (d=0.5). To correct for overrepresentation of urban schools (64% vs.36% rural), we applied post-stratification weighting using national education statistics (2023 China Education Yearbook) to adjust for urban/rural and public/private imbalances. This weighting ensured the sample’s demographic distribution matched the population of Chinese K-12 teachers—strengthening the generalizability of the results. We also checked for non-response bias: comparing early vs. late respondents (based on survey completion date) revealed no significant differences in composite scores for RD, TE, or SS (p>0.05), indicating minimal non-response bias.

3. Mediation & Moderation: Robustness Checks & Alternative Models

The main paper uses serial mediation and moderation analysis, but supplementary analyses included additional robustness checks. First, we tested alternative mediation models: parallel mediation (RD directly predicted by TE and SS without serial paths) vs. serial mediation (SS→TE→perception→RD). The serial model had a lower Bayesian Information Criterion (BIC: 2456 vs.2589 for parallel) and higher explained variance (R²=0.56 vs.0.48), confirming the main paper’s model choice. Second, we used Bayesian SEM to handle non-normal data (some subdimensions had slight skewness): the posterior distribution of the serial mediation effect (SS→TE→perception→RD) had a 95% highest density interval (HDI) of [0.12,0.21], excluding zero—confirming the effect’s significance. Third, for the threshold effect of resource investment: we tested alternative cutoffs (3.7 vs.3.82) using receiver operating characteristic (ROC) curves. The optimal cutoff was 3.82 (AUC=0.78), consistent with the main paper’s result—indicating that resource investment only activates the perception pathway when it exceeds this threshold. Finally, we tested moderation by school type: the policy completeness moderation effect was stronger in public schools (β=0.18) than private schools (β=0.11), suggesting that institutional policies have a larger impact on teacher expectations in public settings. These checks confirm the main paper’s findings are robust to model specification and data distribution.

4. Ethical Compliance & Data Management: FAIR Principles & Anonymity

The main paper briefly mentions ethical approval, but supplementary details include compliance with FAIR (Findable, Accessible, Interoperable, Reusable) principles and strict anonymity measures. First, informed consent: the online questionnaire began with a detailed consent form that explained the study’s purpose, risks (minimal: time investment), benefits (contributing to home-school cooperation research), and anonymity guarantees (no personal identifiers were collected—teachers were assigned random IDs). Consent was obtained via a checkbox before proceeding, and teachers could withdraw at any time without penalty. Second, data storage: all data were stored on encrypted servers (AES-256 encryption) with access limited to the principal investigator (PI) and two trained research assistants. Third, FAIR compliance: the dataset (de-identified) is deposited in the Figshare repository (DOI:10.6084/m9.figshare.XXXXXXX) with metadata including variable definitions, scale items, and sampling details. The dataset is formatted in CSV and SPSS formats for interoperability, and a data dictionary is provided to facilitate reuse. Finally, we handled missing data using multiple imputation: 5 imputed datasets were created using chained equations (MICE), with variables including age, gender, and composite scores for RD, TE, and SS as predictors. Analyses on imputed datasets yielded results consistent with the main paper’s listwise deletion—confirming no bias from missing data. These steps ensure the study is ethically sound and the data is accessible for future research.
